# The Truth about Poisoning with Furs

**Published:** 1924-01

**Authors:** 


### THE TRUTH ABOUT POISONING WITH FURS.

THE recent scare-mongering about poisoned furs, which has caused a flutter in the fur market and has been the subject of many exclamatory head lines, can be put an end to now that the mischief has been tracked to its source. It is ursol, *alias* para-phenylendiamin which is used in the dyeing of almost every kind of fur, expensive or cheap. Ursol itself is colourless, but in contact with an oxidising agent, such as hydrogen peroxide, an insoluble coloured matter, known as Bandrowski's base, is formed. The value of this dye lies in the facts that it gives the fur a smooth sheen, and that the dyeing can proceed in cold water and not at the high temperature injurious to furs which certain other dyes require.

### How it is Done.

Ursol is not used in the dyeing of clothing, but as a fur dye it is pre-eminent, and many tons are manufactured every year. The fur to be dyed is put into the colourless ursol solution and then into a solution of hydrogen peroxide, in which solution the fur turns gradually dark. All the Bandrowski's base which is not linked to the fur, and all the ursol which has not been oxidised to Bandrowski's base are washed away in running water, the fur being put into revolving drums through which water percolates, and in which there are sand and sawdust to help clean the fur. This constant irrigation is kept up for seven or eight hours in properly conducted factories, the water at last coming away from the drums perfectly clear. Investigations have shown that while Bandrowski's base is perfectly harmless, and can be eaten in large doses by experimental animals with impunity, the parent substance, ursol, is very poisonous and capable of producing severe inflammation of the skin, even in minute doses, in susceptible persons. When a lady develops a severe rash on her neck after wearing a fur next to the skin in wet weather, the cause of the trouble is to be sought in faulty washing of the fur directly after it was dyed.

### The Peccant Factories.

The reason why there has of late been such an epidemic of cases of fur poisoning is probably to be sought in careless dyeing and attempts to cheapen the process of rinsing the newly dyed furs. A considerable economy can, of course, be made by perfunctory rinsing with water, and the only course to be adopted with badly rinsed furs is to send them back to the factory whence they came. It is a curious fact that some persons are much more susceptible than others to ursol dermatitis. In a case recently observed a lady had worn a fox stole for some years with impunity before she sent it to be dyed with ursol. When it came back from the factory it provoked a severe rash on the neck after it had been worn for only a few hours, but half a score of other persons, who wore the same fur for several hours, were not at all inconvenienced by it. The number of cases of ursol poisoning is negligible considering the extent to which this dye is used, and there is no reason why the sale of ursol should be forbidden or an edict promulgated against converting the inglorious cat or rabbit into the resplendent sable.

---

**A Revised "Medical Who's Who."**

The Grafton Publishing Co., Ltd., of Chichester House' Chancery Lane, W.C. 2, will publish early next year the 7th Edition of the *Medical Who's Who*, the issue of which was suspended in 1918 on the death of its late editor. Forms for particulars (which were first submitted to the General Medical Council) have been sent to everyone on the *Medical Register*, and the Council is availing itself of the opportunity to convey an official reminder to all practitioners. The revised book will combine the features of the *Medical Register*, the ordinary medical directories and *Who's Who*. It has the endorsement of the highest official medical authority, and its cover will, by permission, be a reproduction of that of the *Register*. The pre-publication price of 25s. post free will be raised to 30s. on publication.

